# The ‘barcode sign’ seen on optical coherence tomography of extensive corneal vascularization

**DOI:** 10.1038/s41433-023-02602-z

**Published:** 2023-07-31

**Authors:** Farida Omar El Zawahry, Frederick Beer, Burcin Kepez Yildiz, Dalia Galal Said, Harminder Singh Dua

**Affiliations:** University of Nottingham and Nottingham University Hospitals NHS Trust, Nottingham, UK

**Keywords:** Eye manifestations, Eye diseases

Optical coherence tomography (OCT) is used extensively in imaging the cornea in health and disease. OCT delineates interfaces of differing refractive indices, which often but not always correspond to histological features of the tissue. Interpretation of images in the context of histology can be difficult. Some tissues or substances, like cyanoacrylate glue on the cornea, obstruct the passage of light and cast shadows obscuring all features deeper to the obstruction [1].

We report a clinical sign, seen on corneal OCT images, which is consistently present in relation to corneal vascularization. Five patients with extensive corneal vascularization secondary to infectious keratitis (2), limbal stem cell deficiency (LSCD) (2) and interstitial keratitis (1) were examined clinically by slit lamp examination and by OCT as part of their routine assessment. The infectious keratitis cases and the interstitial keratitis case were in the healing stage after intensive antibiotic and topical steroid treatment respectively. The LSCD case was 2 years post chemical injury.

**Fig. 1:**
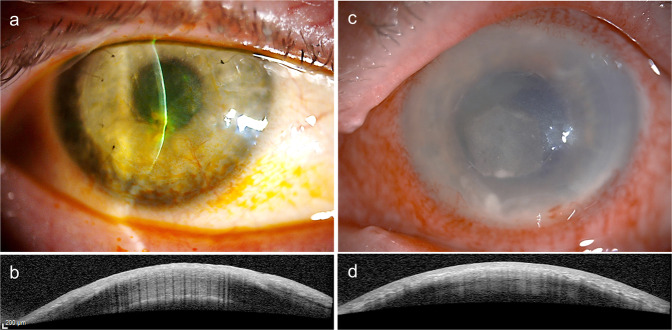
Slitlamp diffuse illumination images and Optical Coherence tomography images of vascularised corneas. **a** Slit lamp photomicrograph of a healing fungal keratitis lesion with extensive feeder vessels. **b** Anterior segment optical coherence tomography (OCT) of peripheral corneal vessels demonstrating a characteristic ‘barcode sign’ seen as vertical dark lines in the OCT scan. **c** Slit lamp photomicrograph of a cornea with healing bacterial keratitis associated with extensive vascularization. **d** Anterior segment OCT image illustrating the ‘barcode sign’ seen as vertical dark lines.

Anterior segment (AS) OCT was carried out with a Heidelberg Spectralis (Spectralis HRA + OCT, Heidelberg, Germany) with the light beam at right angles to the major trunks of the corneal vessels giving cross-sectional images of the cornea. Twenty-one scans from limbus to the center of the superior and inferior halves, of the cornea were obtained. All forty-two scans were examined individually for each patient and correlated with the slit lamp examination and images using (Topcon slit lamp SL-D701, Tokyo, Japan). Each major vascular trunk (400 µ–1200 µ), both afferent and efferent, with an active circulation obstructed the passage of light completely. A dark shadow, starting from the depth of location of the vessels and extending the entire thickness of the cornea posterior to the location, was seen as a straight line on the OCT scan. The shadow was well delineated as a hypo-reflective stripe in the general hyper-reflective background of the entire image. The width of the shadow corresponded to the width of the vessel that cast the shadow. In eyes with extensive vascularization with multiple vessels, several vertical lines of the shadows cast were visible in a single scan, giving it the appearance of a bar code, hence the descriptor, the ‘bar code sign’ is proposed for this appearance (Fig. 1).

Though arteries (afferent) and veins (efferent) have different vessel-wall structures, this is not the same as afferents and efferents of corneal neovascularization. As both type of vessels produced an equally dense shadow, it is likely that the obstruction to the passage of light was by the column of circulating blood in the vessels rather than the vessel wall. Moreover, ghost vessels that did not contain a blood column, did not cast a shadow. The corneal stroma has a rich network of nerves, especially anteriorly. These were not apparent on OCT imaging though in-vivo confocal microscopy imaging and whole mount staining of nerves of diseased corneas show extensive thickening of nerves [2, 3]. The bar-code sign appears to be specific to corneal vessels with active circulation.
